# The impact of different autoclaving cycles on the properties of rotary endodontic files: an in vitro study

**DOI:** 10.1186/s12903-026-09883-2

**Published:** 2026-11-05

**Authors:** Mohammed A. Youssef, Dina S. Farahat, El-Sayed Gad Eid

**Affiliations:** 1 Department of Dental Biomaterials, Faculty of Oral and Dental Medicine, Delta University for Science and Technology, International Coastal Rd, Gamasa, Dakahleya, Egypt; 2 Department of Dental Biomaterials, Faculty of Dentistry, Mansoura University, El-Gomhouria Street, Mansoura, 35516 Egypt

**Keywords:** NiTi rotary files, Autoclaving, Cyclic fatigue resistance, Surface roughness, Root canal dentine roughness

## Abstract

**Background:**

Whether autoclaving affects mechanical and surface characteristics of nickel–titanium rotary instruments is a subject of debate. The present in vitro investigation aimed to determine how autoclaving influences cyclic fatigue resistance and surface roughness of three NiTi rotary files — ProTaper Ultimate (PTU), TruNatomy (TN), and ProTaper Next (PTN), and to assess post-instrumentation root canal dentine roughness.

**Methods:**

A total of 180 rotary files were allocated into 3 groups (*n* = 60) based on file type, and each group was further divided into three subgroups (*n* = 20) depending on the number of autoclaving cycles applied (0, 5, and 10). Following sterilization, 10 files per subgroup underwent cyclic fatigue testing in a custom-fabricated apparatus with artificial canals. The remaining 10 files were subjected to surface roughness evaluation via optical profilometry. To assess post-instrumentation dentine roughness, 90 lower premolars were prepared, sectioned longitudinally, and examined using scanning electron microscopy (SEM). SEM micrographs were processed through image-analysis software for roughness quantification. Statistical comparisons were performed using two-way and three-way ANOVA at α = 0.05.

**Results:**

Cyclic fatigue resistance of TN declined significantly after exposure to 5 and 10 autoclaving cycles, whereas PTU and PTN demonstrated resistance to such changes. Among the three systems, TN recorded the highest cyclic fatigue resistance, followed by PTU and PTN. Surface roughness increased significantly only for TN with progressive autoclavingwhile it increased for PTU and PTN only after exposure to 10 and 5 cycles, respectively. At zero autoclaving cycles, TN exhibited the least roughness, with no statistically significant differences between PTU and PTN. After 5 and 10 cycles, PTN showed the highest roughness, which differed significantly from the other file systems. Regarding dentine roughness, autoclaving of PTU caused an increase in roughness in most subgroups, whereas autoclaving of TN increased dentine roughness in apical and coronal thirds after 10 cycles. In contrast, for PTN, dentine roughness significantly decreased in most subgroups after autoclaving.

**Conclusion:**

The effect of repeated autoclaving in this study was dependent on the file-system type. Clinicians should consider file-specific responses to autoclaving when establishing sterilization and reuse protocols to optimize instrument safety and root canal surface quality.

## Background

Root canal therapy encompasses multiple sequential steps aimed at eliminating microbial contamination and devitalized pulp tissue to promote long-term healing outcomes. A fundamental stage is root canal preparation, which involves the mechanical cleaning and shaping of the canal system in preparation for obturation. Nevertheless, this phase is complicated by the inherent variability and complexity of root canal anatomy, which has driven the development of numerous instrument designs and clinical techniques [1]. Historically, stainless steel hand files were the primary tools used for this purpose, offering high yield and ultimate mechanical strength [2]. Their application in curved canals, however, was frequently associated with procedural errors including ledges, perforations, canal transportation, and zipping [3, 4]. Furthermore, precurving stainless-steel instruments to accommodate curved anatomy is impractical in rotational motion [5].

The adoption of motorized rotary instrumentation has considerably improved procedural efficiency, reducing overall treatment time [3]. The introduction of nickel-titanium (NiTi) instruments by Walia et al. [6] in 1988 marked a transformative development in endodontic practice. These instruments exhibit superior flexibility, excellent cyclic fatigue resistance, and enhanced mechanical performance relative to their stainless-steel counterparts [7]. The superelastic and shape-memory behavior of NiTi alloys is well recognized; nonetheless, these files remain susceptible to unexpected fracture resulting from cyclic fatigue or torsional overload during clinical use [8]. Cyclic fatigue arises from repeated cycles of compressive and tensile stresses at the point of highest flexure, occurring in the absence of visible plastic deformation [9]. The fracture resistance of NiTi files is governed by multiple factors including alloy composition, manufacturing methodology, and instrument geometry [10]. Proprietary heat, mechanical, and surface treatments were developed to optimize the mechanical performance and enhance NiTi alloys’ resistance to fracture [11].

Instrument geometry constitutes a key determinant of fracture resistance, encompassing dimensions such as size, taper, flute length, and cross-sectional morphology [12]. The cross-sectional profile dictates the contact area between the instrument and root canal walls; a greater contact area correlates with increased dentine removal per rotation. Zhang et al. [13] demonstrated that cross-sectional geometry exerted a more substantial influence on stress distribution under torsional and bending loads than either taper or instrument size.

Characterization of the surface roughness (SR) of NiTi rotary instruments yields valuable data regarding surface integrity, clinical performance, and potential limitations, with direct implications for the mechanical properties of instrumented dentine, including hardness and elastic behaviour [14]. The SR of the cutting blade specifically influences several clinically relevant mechanical properties. Elevated surface roughness raises friction at the file-dentine interface, thereby compromising efficiency of cutting, cyclic fatigue resistance, and overall safety [15]. Consequently, surface irregularities may serve as initiation sites for stress concentration, crack propagation, and eventual instrument fracture in clinical procedures [16].

Beyond shaping, obturation is a determinant of endodontic treatment success. Increased dentinal wall roughness may facilitate improved mechanical interlocking between dentine and sealer, potentially enhancing the quality of obturation through improved adhesion and sealer penetration [17]. Conversely, rough root canal surfaces may promote bacterial adherence and colonization [18]. Multiple methodologies have been employed to assess root canal dentine roughness, including atomic force microscopy (AFM) and scanning electron microscopy (SEM) [19].

NiTi rotary instruments are routinely reused in clinics for cost-effectiveness, necessitating multiple autoclave cycles to prevent cross-infection between patients [20]. Given that thermal processing is integral to the manufacturing of NiTi files, the temperatures encountered during sterilization may potentially alter the mechanical properties of these instruments [21]. Although the impact of autoclaving on the mechanical and physical properties of rotary endodontic files has been investigated, the literature remains inconclusive regarding whether sterilization induces clinically significant deterioration of these properties [22].

The aim of this investigation was to examine the influence of varying numbers of autoclaving cycles on the properties of three NiTi rotary file systems. The first null hypothesis stated that file type would have no bearing on cyclic fatigue resistance, file surface roughness, or post-instrumentation dentine roughness. The second null hypothesis stated that autoclaving would exert no significant effect on any of the aforementioned parameters.

## Materials and methods

### Materials

The types, manufacturers, lot numbers, and characteristic features of the files used in this study are shown in Table 1.

**Table 1:** Files used in the study

File	Manufacturer	Size	Lot number	Characteristic feature
Protaper Ultimate (PTU)	Dentsply Sirona-Johnson City, Tennessee, USA	25- taper 8 (F2)	0000392168 − 0000305501	Alternating offset machining
Trunatomy (TN)		26- taper 4 (prime)	0000384644 − 0000305501	Off-centred parallelogram cross section- thin wire
Protaper Next (PTN)		25- taper 6 (X2)	0000314429–0000376218	M- wire off-centred rectangular cross section

### Sample size calculation

Sample size was calculated based on previous research by Ali et al. [23] using the G*Power program version 3.1.9.7 to calculate sample size based on an effect size of 1.6, error (α) = 0.05, and power (1-β) = 0.95. The calculated sample size was 4 in each subgroup. The sample size was increased to 10 for each group to enhance the reliability and robustness of the results.

### Study design

A total number of 180 endodontic rotary files (size 25) was used in this study and randomly allocated into 3 groups (*n* = 60) according to the type of file: Group A: Protaper Ultimate) (PTU (, Group B: Trunatomy (TN (, Group C: Protaper Next (PTN). Each group was subdivided into 3 subgroups (*n* = 20) according to autoclaving cycles to which the files were subjected. Subgroup 1: 0 autoclaving cycles, Subgroup 2: 5 autoclaving cycles and Subgroup 3: 10 autoclaving cycles. Ten files from each subgroup were used for cyclic fatigue testing, while the remaining ten files were used to evaluate the files’ surface roughness and then used to assess the resulting dentine roughness following instrumentation.

### Cyclic fatigue resistance testing

A total number of 90 rotary files were used. 30 files from each type of the rotary files (PTU, TN and PTN) were selected and inspected for manufacturing defects using a stereomicroscope at 20× magnification (Olympus, Shinjuku, Tokyo, Japan) [24]. The files exhibiting visible manufacturing defects or deformities were excluded. The files from each type were further divided into 3 subgroups (*n* = 10) according to the number of autoclaving cycles: the first subgroup was subjected to 0 cycles, the second to 5, and the third to 10. The selected rotary files underwent sterilization using a standard autoclave (Tuttnauer, USA) set at 121 °C and 15 psi for 15 min per cycle. The instruments were allowed to dry and cool to room temperature [25].

**Fig. 1:**
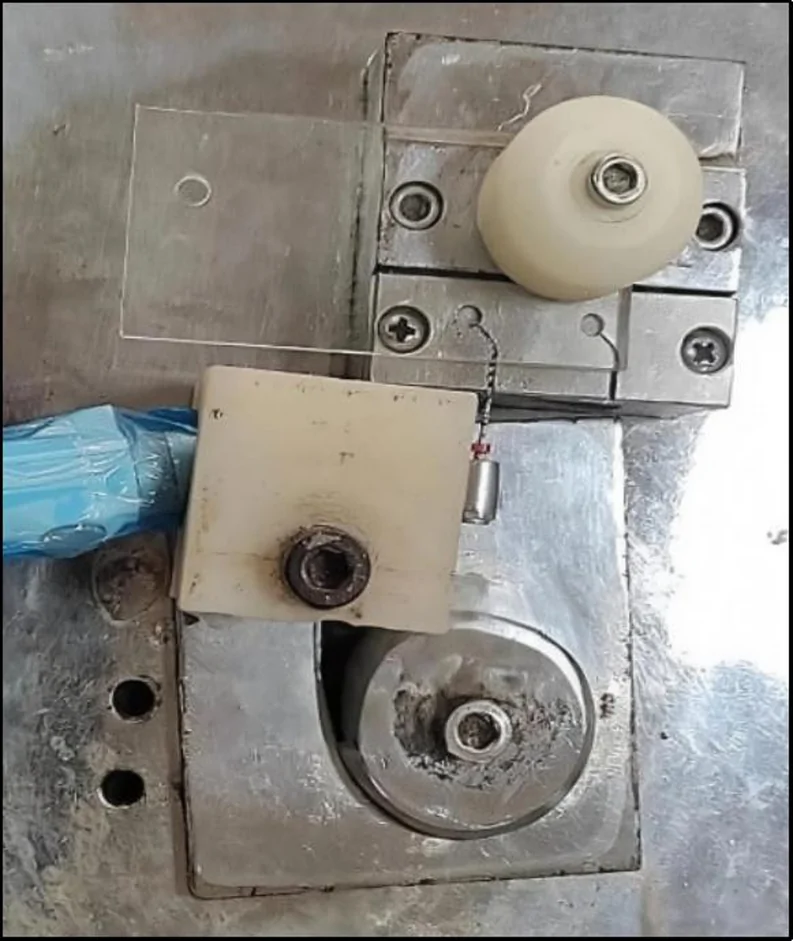
Custom-made device used in cyclic fatigue testing

To evaluate cyclic fatigue resistance, the number of cycles to fracture was measured to give an indication of a file’s resistance to cyclic fatigue. A custom designed device was used, as shown in Fig. 1. It consisted of a frame supporting stainless-steel block containing the artificial root canals. Each canal had a 45º angle and a 5 mm radius of curvature. The centre of curvature was 5 mm from the instrument tip [26]. The files were rotated within the canals at the speed and torque recommended by the manufacturer till fracture. The time to fracture (TTF) in seconds was recorded using a stopwatch. Then, the number of cycles to fracture (NCF) was calculated using the following equation [27]:

$$\begin{aligned}\\&\mathrm{NCF}=\mathrm{Time}\;\mathrm{to}\;\mathrm{fracture}\;\mathrm{in}\\&\;\mathrm{seconds}\;\times\;\mathrm{the}\;\mathrm{recommended}\\&\;\mathrm{rotational}\;\mathrm{speed}\;\mathrm{for}\;\mathrm{each}\;\mathrm{file}\;/\;60\end{aligned}$$

### Surface roughness assessment of the investigated files

A total number of 90 endodontic files were used. 30 files from each type were selected and inspected for manufacturing defects as previously mentioned. The files from each type were further divided into 3 subgroups (*n* = 10) according to the number of autoclaving cycles: the first subgroup was subjected to 0 cycles, the second to 5, and the third to 10 cycles. The selected rotary files underwent sterilization using a standard autoclave (Tuttnauer, USA) set at 121 °C and 15 psi for 15 min per cycle. Then, the surfaces topography and roughness were analysed for each subgroup using a 3D non-contact optical profilometer (Zygo-Maxim GP 200 profilometer).

**Fig. 2:**
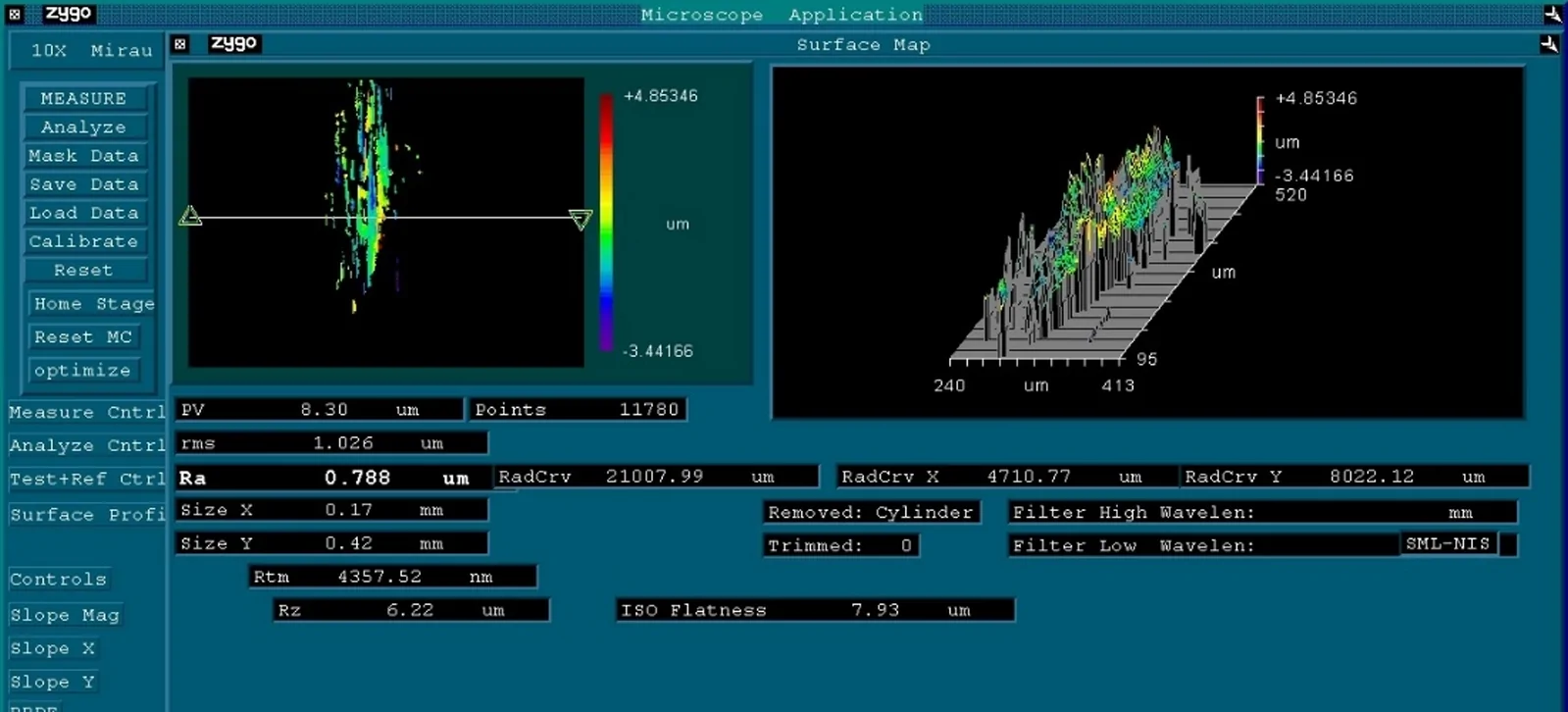
Image analysis of a non-autoclaved PTU file

As proposed by Ferreira et al. [28], the scanning areas (211 × 211 μm) were located 3 mm coronally from the instrument tip. The surface areas of the cutting blade and the flute of the instrument were assessed three times for each subgroup and the combined average roughness value (Ra) (the arithmetic mean of the height of peaks and depth of valleys from a mean line) was calculated using advanced texture analysis software **(**MetroPro™ software, ZYGO Corporation, Middlefield, CT, USA) as shown in Fig. 2 [29]. 

### Assessment of post instrumentation root canal dentine roughness

A total of 90 intact human mandibular single rooted premolars extracted for orthodontic purposes were used. The tissue remnants on root surfaces were cleaned using an ultrasonic scaler (Woodpecker UDS-P LED, Guilin Woodpecker Medical Instrument Co., LTD, Guilin, China). Then, all teeth were immersed in thymol for 24 h and stored in saline till use. The premolars were instrumented using the same files after autoclaving and assessment of their roughness in the previous test. The premolars were divided into three groups (*n* = 30) according to the type of files used, then subdivided into 3 subgroups (*n* = 10) according to the number of autoclaving cycles to which the files were subjected. Group 1 was instrumented with PTU files. Group 2 was instrumented with TN files, while Group 3 was instrumented with PTN files. After mounting in acrylic blocks, the prepared roots were sectioned longitudinally through the root canals in a bucco-lingual direction using a low-speed diamond saw (Pico 155 Pace Technologies, Tucson, AZ, USA) under water cooling, resulting in two equal root halves.

A total of 90 halves (one half from each premolar) were chosen to be examined for root canal dentine roughness under a scanning electron microscope (JEOL JSM-6510LV, JEOL Ltd., Akishima, Tokyo, Japan) after gold plating was performed using a sputter coater (SPI Sputter Coater, SPI Supplies, West Chester, PA, USA). Three images were taken under magnification of 1000 x and a 10 μm scale bar under identical imaging conditions for each specimen for the cervical, middle and apical thirds of each root. The images were analyzed on an Intel^®^ Core i7^®^ based computer using VideoTest Morphology^®^ software (VideoTesT, Ltd., St Petersburg, Russia) with a specific built-in routine for area, % area, object counting, contact angle, surface waviness, and roughness analysis, as well as ImageJ software with Surf Char J plugin (ImageJ 154-win-java8, NIH, Bethesda, USA).

**Fig. 3:**
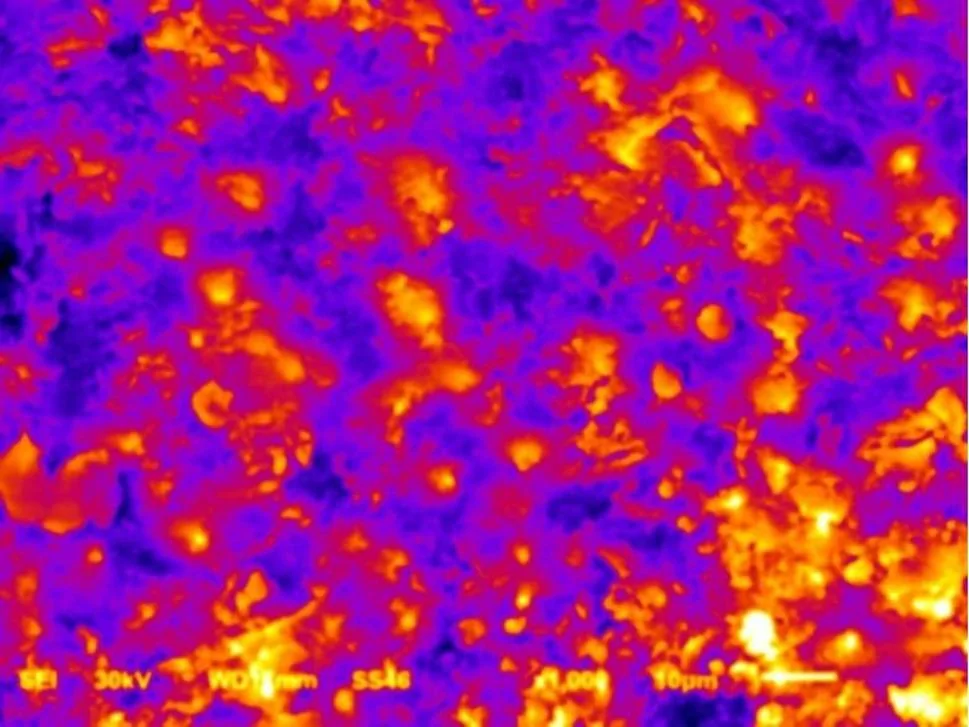
Waviness profile generation of an SEM image

**Fig. 4:**
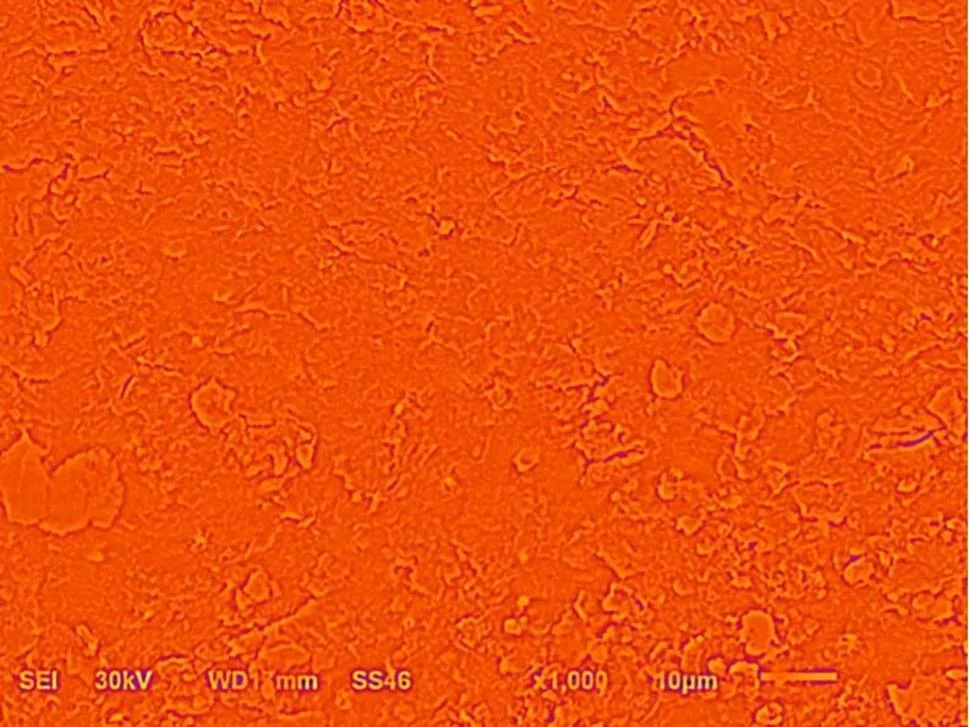
Roughness image obtained by subtracting the waviness profile from the original micrograph

SEM images were converted to 32-bit grayscale prior to analysis. Surface waviness and roughness components were separated using the waviness roughness plugin, which applies a Gaussian blur filter to generate the waviness profile (Fig. 3). The roughness image was subsequently obtained by subtracting the waviness component from the original image, thereby isolating the short-wavelength surface irregularities. A Gaussian cut-off value unit of (4 μm) was applied consistently to all images following preliminary optimization to ensure uniform separation of waviness and roughness across all experimental groups (Fig. 4). The resulting roughness images were coded prior to analysis to blind the examiner to the experimental group, then analyzed using a standardized Roughness Calculation plugin in ImageJ with identical magnification, region-of-interest selection, and image-processing procedures for all specimens to calculate the arithmetic mean surface roughness (Ra) using a minimum sampling length of 4 μm over the whole image to obtain repeatable and reproducible results [30, 31]. The three SEM images obtained from each specimen were analyzed separately, then the average roughness was calculated for each specimen.

### Statistical analysis

The normality of data distribution was assessed using the Shapiro-Wilk test. Data were statistically analysed using two-way and three-way analyses of variance (ANOVA) to assess the effects of file type, number of autoclaving cycles, and root canal region on cyclic fatigue resistance and surface roughness. Post-hoc multiple comparisons were conducted using Tukey’s test to identify specific group differences when significant main or interaction effects were detected. The data were expressed as mean ± standard deviation (SD). A significance level of *p* < 0.05 was considered statistically significant.

## Results

### Cyclic fatigue resistance

**Fig. 5:**
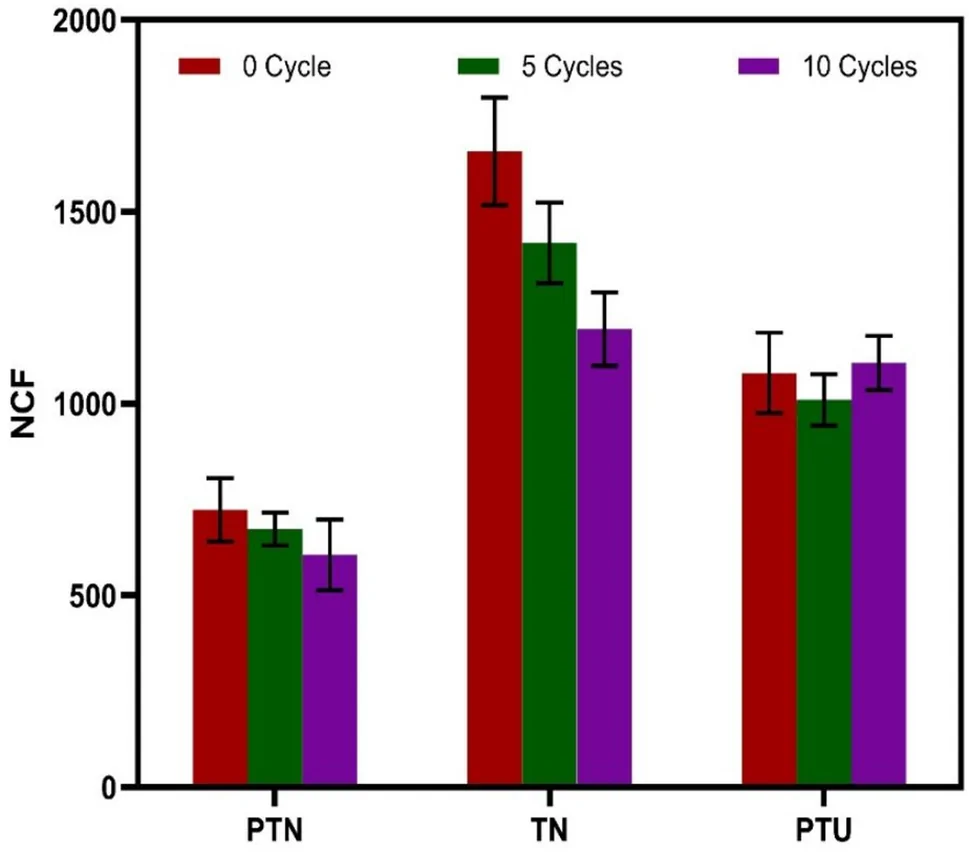
Means and SDs of cyclic fatigue resistance of different rotary files subjected to different numbers of autoclaving cycles

The means and standard deviations of the number of cycles till fracture (cyclic fatigue resistance are shown in Table 2. Two-way ANOVA results (Table 3) indicated that the rotary file type, the number of autoclaving cycles, and the interaction between the two variables had a significant effect on the cyclic fatigue resistance (*P* < 0.001), as shown in Table 2. Tukey’s test showed significant differences in cyclic fatigue resistance among the different files. TN files showed the highest cyclic fatigue resistance after 0 and 5 autoclaving cycles, while PTN exhibited the lowest cyclic fatigue resistance among the studied groups (*p* < 0.001) at all cycle numbers. The number of autoclaving cycles had no significant effect on the cyclic fatigue of the files, except for TN files, in which autoclaving induced a significant decrease in cyclic fatigue resistance with increasing numbers of cycles from zero to five (*p* < 0.001) and from five to ten (*p* < 0.001), as shown in Table 2; Fig. 5.

**Table 2:** Means ± SDs of the number of cycles till fracture (NCF) (cyclic fatigue resistance) among the studied groups

Groups	0 Cycles	5 Cycles	10 Cycles
PTN	723 ± 82.53 ^Aa^	673 ± 42.91 ^Aa^	606 ± 91.93 ^Aa^
TN	1658 ± 140.2^Cc^	1419 ± 104.9^Cb^	1194 ± 95.71^Ba^
PTU	1080 ± 105.3^Ba^	1010 ± 66.69^Ba^	1106 ± 70.96^Ba^

Different upper−case letters indicate significant differences in cyclic fatigue resistance among different types of files subjected to the same number of autoclaving cycles (columns)

** Different lower−case letters indicate significant differences in cyclic fatigue resistance in the same type of file subjected to different numbers of autoclaving cycles (rows). (*p *≤ 0.05)

**Table 3:** Two-way ANOVA for the cyclic fatigue resistance (NCF) based on the file type and autoclaving cycles

Source	Type III Sum of Squares	df	Mean Square	F	*p*-value	Partial Eta Squared
Corrected Model	9784317.520	8	1223039.690	142.012	**<** 0.001	0.933
Intercept	99575378.765	1	99575378.765	11562.101	< 0.001	0.993
File type	8591369.996	2	4295684.998	498.789	< 0.001	0.925
Autoclaving	526644.323	2	263322.161	30.575	< 0.001	0.430
File type x Autoclaving	666303.200	4	166575.800	19.342	< 0.001	0.489
Error	697589.965	81	8612.222			
Total	110057286.250	90				
Corrected Total	10481907.485	89				

### Surface roughness

**Fig. 6:**
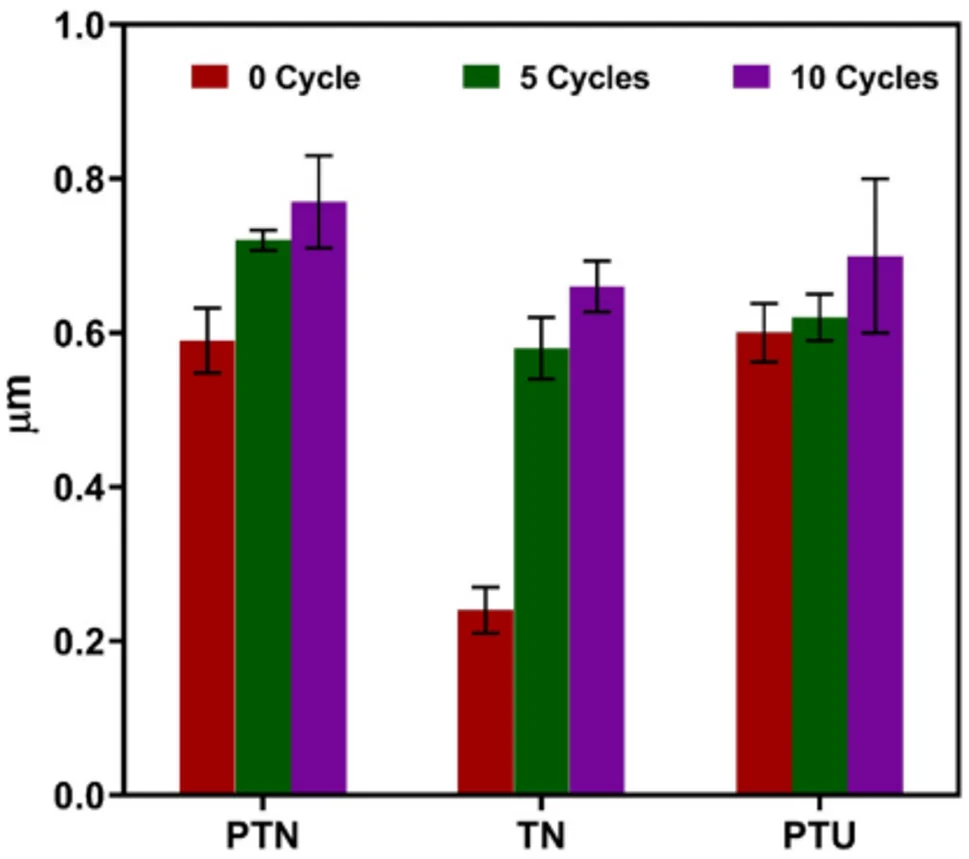
Means **±** SD of surface roughness (µm) for different endodontic files subjected to different numbers of autoclaving cycles

The means and standard deviations of files’ surface roughness (µm) are shown in Table 4; Fig. 6. The results of the two-way ANOVA (Table 5) indicated that the rotary file type, the number of autoclaving cycles, and their interaction had a significant effect on the files’ surface roughness (*p* < 0.001). The results of post hoc Tukey tests showed that at zero autoclaving cycles, TN exhibited significantly lower surface roughness than the other files (*p* < 0.001). However, there was no significant difference between PTU and PTN (*p* = 1). At 5 and 10 autoclaving cycles, PTN was found to be the roughest file, as it was significantly rougher than TN (*p* < 0.001) and PTU (*p* = 0.001 with 5 cycles and *p* = 0.02 with 10 cycles). Nevertheless, no statistically significant differences were found between PTU and TN (*p* = 0.762 with 5 cycles and *p* = 0.669 with 10 cycles). Only TN surface roughness was affected by autoclaving at both 5 and 10 cycles among the files evaluated in the present study. (*p* < 0.0001). A non-significant increase in roughness was noted in PTU when increasing autoclaving cycles from zero to five (*p* = 0.75). However, the roughness significantly increased when autoclaving cycles increased from five to ten (*p* = 0.011). A significant rise in surface roughness of PTN was reported with increasing autoclaving cycles from zero to five (*p* < 0.001), nevertheless, no significant increase in roughness occurred when autoclaving cycles were increased from five to ten (*p* = 0.145).

**Table 4:** Mean values ± SDs of file roughness (µm) among the studied groups

Groups	0 Cycles	5 Cycles	10 Cycles
PTN	0.59 ± 0.036 ^Ba^	0.72 ± 0.013 ^Bb^	0.773 ± 0.06 ^Bb^
TN	0.24 ± 0.03 ^Aa^	0.58 ± 0.04 ^Ab^	0.66 ± 0.033 ^Ac^
PTU	0.60 ± 0.038 ^Ba^	0.62 ± 0.03 ^Aa^	0.7 ± 0.1^Ab^

*Different uppercase letters indicate statistically significant differences among different types of rotary files (columns)

**Different lowercase letters indicate statistically significant differences between the number of autoclaving cycles (rows) (Tukey’s post-hoc test, *p* < 0.05)

**Table 5:** Two-way ANOVA for file roughness (µm) based on the file type and autoclaving cycles

Source	Type III Sum of Squares	df	Mean Square	F	*p*-value	Partial Eta Squared
Corrected Model	1.869	8	0.234	102.877	**<** 0.001	0.910
Intercept	33.495	1	33.495	14746.667	**<** 0.001	0.995
File type	0.639	2	0.319	140.634	**<** 0.001	0.776
Autoclaving	0.854	2	0.427	187.915	**<** 0.001	0.823
File type x Autoclaving	0.377	4	0.094	41.480	**<** 0.001	0.672
Error	0.184	81	0.002			
Total	35.549	90				
Corrected Total	2.053	89				

### Root canal dentine roughness

**Fig. 7:**
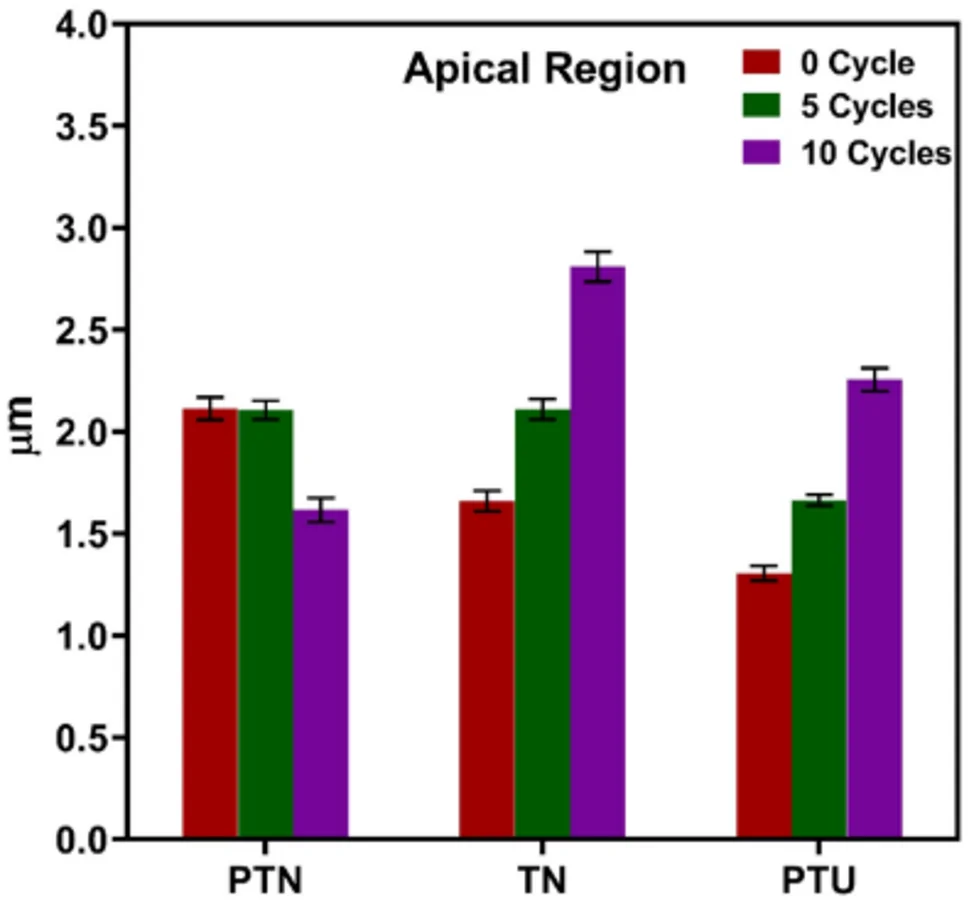
Means and standard deviations of post-instrumentation dentine roughness with different files and different numbers of autoclaving cycles at apical third

**Fig. 8:**
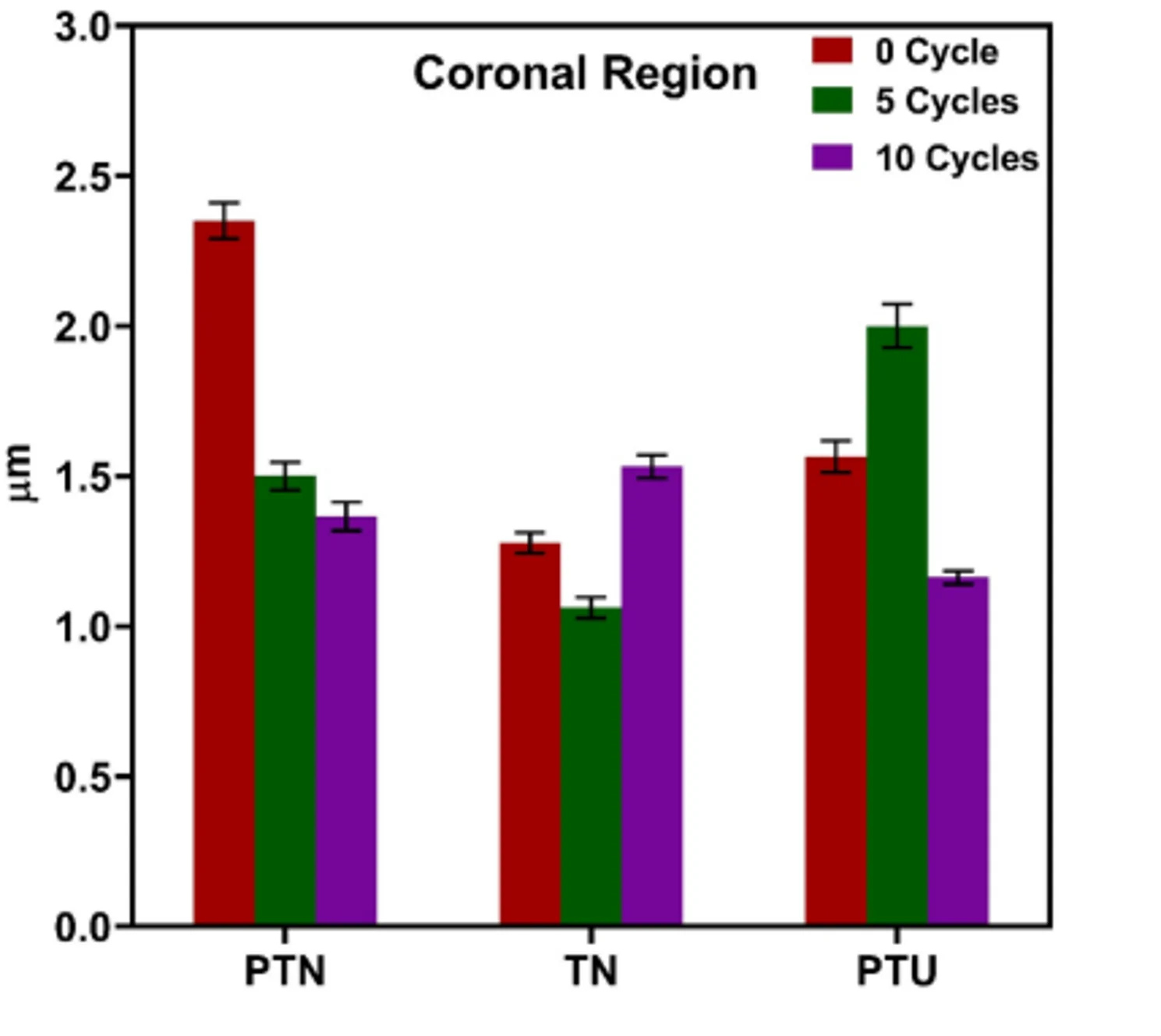
Means and standard deviations of post-instrumentation dentine roughness with different files and different numbers of autoclaving cycles at the coronal third

**Fig. 9:**
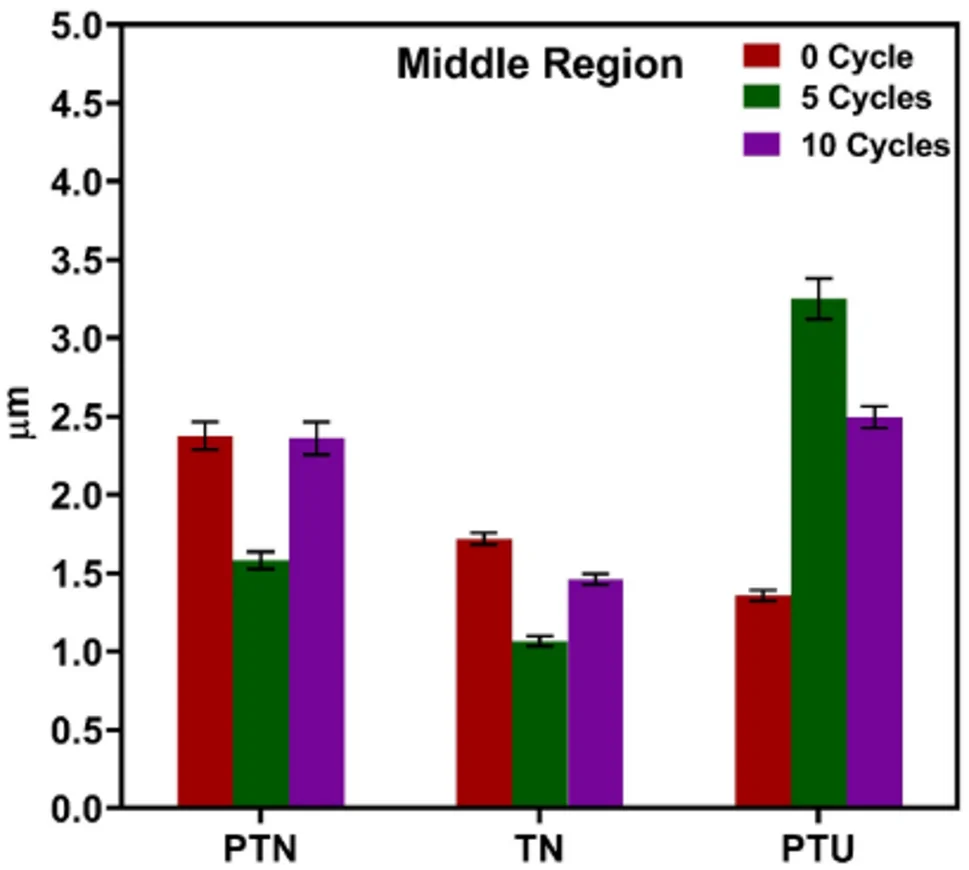
Means and standard deviations of post-instrumentation dentine roughness with different files and different numbers of autoclaving cycles at the middle third

**Fig. 10:**
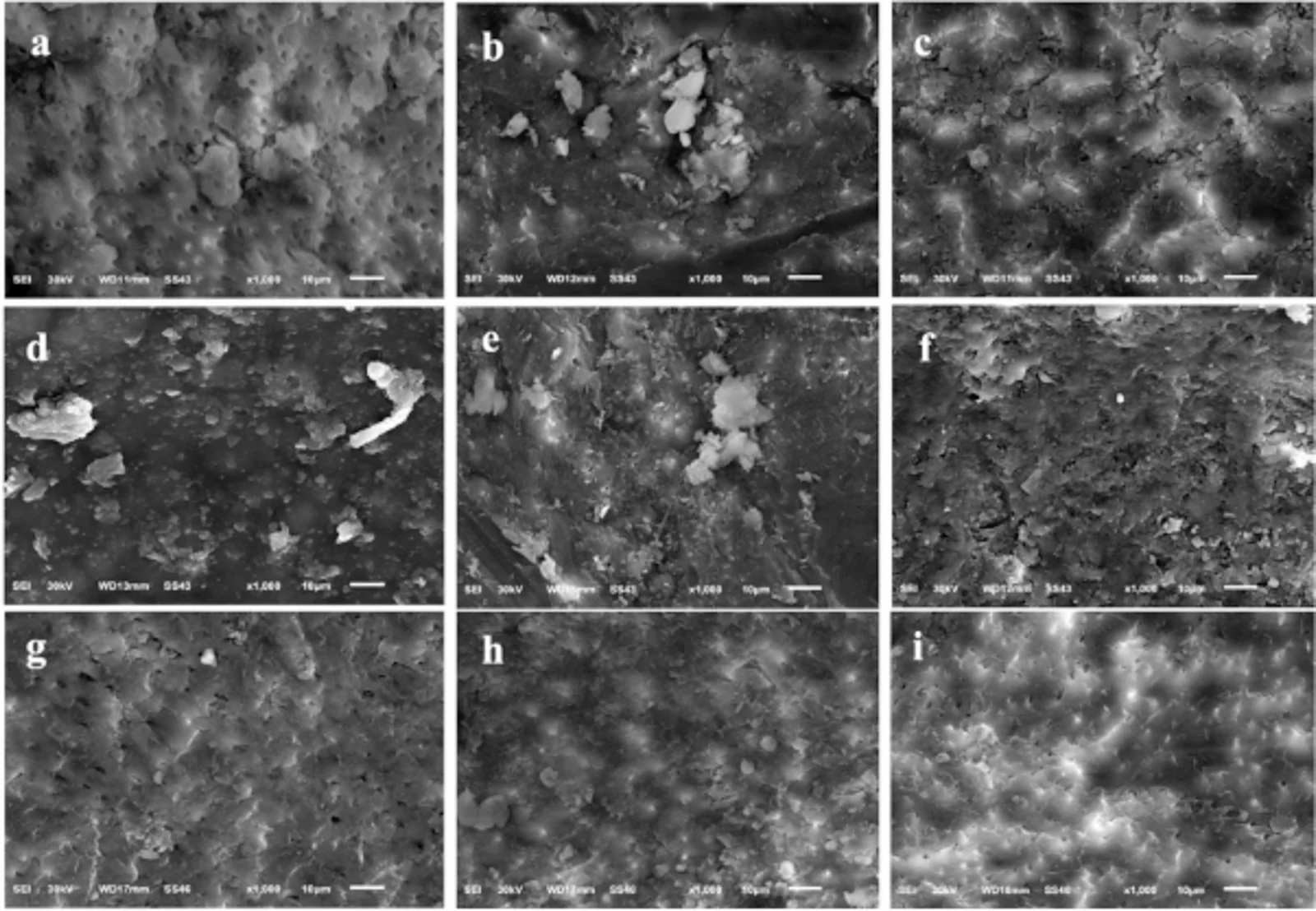
Representative SEM images used for dentin roughness evaluation in apical third of the root; (**a**) PTU at zero cycles, (**b**) PTU at 5 cycles, (**c**) PTU at 10 cycles, (**d**) TN at 0 cycles, (**e**) TN at 5 cycles, (**f**) TN at 10 cycles, (**g**) PTN at 0 cycles, (**h**) PTN at 5 cycles, (**i**) PTN at 10 cycles

The results of post instrumentation root canal dentine roughness are shown in Tables 6 and 7; Figs. 7, 8 and 9. Representative SEM images used to calculate dentin roughness are shown in Fig. 10. Three-way ANOVA results indicated that the rotary file type, the number of autoclaving cycles and root canal region had a significant impact on the root canal dentine roughness test results (*p* < 0.001). The interactions between the file type and the other two variables had a significant effect on root canal dentine roughness, as well as the interaction between autoclaving and root canal region. The interaction between the three variables had a significant effect on the mentioned parameter (*p* < 0.001). Tukey tests showed that for non-autoclaved files, instrumentation resulted in significantly higher surface roughness of the root canal dentine with PTN for all thirds (*p* < 0.001) and the least roughness of root canal dentine with PTU in apical and middle thirds, and TN in coronal thirds (*p* < 0.001). At 5 autoclaving cycles, PTU produced the highest roughness in the middle third, whereas TN produced the least roughness in the coronal and middle thirds (*p* < 0.001). For 10 cycle autoclaved files, TN produced the highest roughness in both apical and coronal thirds, while PTU produced the highest roughness in the middle third. PTU produced the least roughness in coronal thirds, while PTN produced the least roughness in apical third (*p* < 0.001).

**Table 6:** Means ± SDs of dentine roughness (um) among the studied groups

File type	Autoclaving	Apical	Coronal	Middle
PTN	0 Cycle	2.114 ± 0.05553^Cb1^	2.351 ± 0.05918^Cc2^	2.376 ± 0.08874^Cb2^
	5 Cycles	2.107 ± 0.04606^Bb2^	1.500 ± 0.04654^Bb1^	1.581 ± 0.05433^Ba1^
	10 Cycles	1.616 ± 0.06004^Aa2^	1.366 ± 0.04755^Ba1^	2.360 ± 0.1043^Bb3^
TN	0 Cycle	1.659 ± 0.04978^Ba2^	1.278 ± 0.03364^Ab1^	1.719 ± 0.03781^Bc2^
	5 Cycles	2.110 ± 0.04960^Bb2^	1.062 ± 0.03435^Aa1^	1.066 ± 0.03223^Aa1^
	10 Cycles	2.809 ± 0.07353^Cc2^	1.532 ± 0.03834^Cc1^	1.461 ± 0.03616^Ab1^
PTU	0 Cycle	1.305 ± 0.03570^Aa1^	1.565 ± 0.05325^Bb2^	1.356 ± 0.03420^Aa1^
	5 Cycles	1.663 ± 0.02624^Ab1^	2.000 ± 0.07211^Cc2^	3.251 ± 0.1295^Cc3^
	10 Cycles	2.256 ± 0.05636^Bc2^	1.163 ± 0.02246^Aa1^	2.496 ± 0.06973^Cb3^

*Different uppercase letters indicate statistically significant differences among different types of rotary files at the same number of autoclaving cycles (columns)

**Different lowercase letters indicate statistically significant differences in dentine roughness at different numbers of autoclaving cycles for the same type of file (columns)

*** Different numbers indicate different thirds of roots for the same type of file at the same number of autoclaving cycles (rows). (Tukey’s post-hoc test, *p* < 0.05)

**Table 7:** Three-way ANOVA for the file roughness (µm) based on the file type, autoclaving, and the root canal region

Source	Type III Sum of Squares	df	Mean Square	F	*p*-value	Partial Eta Squared
Intercept	890.153	1	890.153	42561.071	< 0.001	0.994
Corrected Model	75.084	26	2.888	138.077	< 0.001	0.937
File type	4.461	2	2.230	106.638	< 0.001	0.467
Autoclaving	0.971	2	0.485	23.203	< 0.001	0.160
Root canal region	10.783	2	5.391	257.784	< 0.001	0.680
File type x Autoclaving	19.202	4	4.800	229.527	< 0.001	0.791
File type x Root canal region	14.461	4	3.615	172.853	< 0.001	0.740
Autoclaving x Root canal region	6.657	4	1.664	79.575	< 0.001	0.567
File type x Autoclaving x Root canal region	16.581	8	2.073	99.101	< 0.001	0.765
Error	5.082	243	0.021			
Total	973.735	270				
Corrected Total	80.166	269				

A significant impact of autoclaving was found on PTU with all thirds’ specimens for which the dentine roughness increased significantly with increasing number of autoclaving cycles from zero to five then from five to ten (*p* < 0.0001), and increased with increasing autoclaving cycles from zero to five significantly for coronal and middle thirds (*p* < 0.0001), then decreased significantly when increasing autoclaving cycles from five to ten (*p* < 0.001).

Also, in TN, a significant rise was noticed in apical dentine roughness with increasing autoclaving cycles from zero to five (*p* < 0.0001) and ten (*p* < 0.0001), while in coronal and middle thirds a significant drop in roughness was noticed with increasing autoclaving cycles from zero to five, followed by a significant rise in roughness with increasing autoclaving cycles from five to ten. With PTN, a significant drop in apical dentine roughness was shown when increasing autoclaving cycles to ten (*p* < 0.001) with non-significant differences between non-autoclaved and 5 cycle autoclaved files (*p* > 0.999), while roughness of coronal dentine decreased progressively with increasing autoclaving cycles from zero to five and from five to ten (*p* < 0.001), and roughness of middle third dentine decreased significantly when increasing autoclaving cycles from zero to five and increased significantly from five to ten cycles.

In this study, significant effects of the different thirds of root on dentine roughness were seen in PTU reporting the highest roughness for coronal third (*p* < 0.001) and middle thirds in the other subgroups (*p* < 0.001). For TN, non-significant differences were seen between apical and middle thirds (*p* = 0.875), while coronal dentine showed the least roughness with non-autoclaved files (*p* < 0.001). With the other two subgroups of TN, the middle third showed the highest roughness (*p* < 0.001), while the apical third showed the least roughness with 5 cycles group and the coronal third showed the least roughness with 10 cycle group (*p* < 0.001). For PTN, the apical dentine showed the least roughness (*p* < 0.001) with non-significant differences between coronal and middle thirds (*p* > 0.999) for the non-autoclaved files, while the apical dentine showed the highest roughness with 5 cycles (*p* < 0.001) and the middle third showed the highest roughness with 10 cycles group (*p* < 0.0001). Overall, the findings suggest that the effect of autoclaving on dentine roughness is dependent on both the instrument type and the root canal region, indicating a complex interaction between these experimental factors and that the influence of autoclaving on dentine roughness varies according to both the file system and the specific root canal region examined.

## Discussion

The present investigation examined the impact of autoclaving on cyclic fatigue resistance, surface roughness, and post-instrumentation dentine roughness of three contemporary NiTi rotary file systems as a critical point in daily use of rotary instruments. Significant intergroup differences were identified for all three outcome measures, leading to the rejection of the first null hypothesis. Additionally, autoclaving had a significant negative impact on the cyclic fatigue resistance of TN and significantly increased the surface roughness of all files after 10 sterilization cycles. Post-instrumentation dentine roughness was also significantly altered for PTU and TN. Accordingly, the second null hypothesis was likewise rejected.

Despite continuous design refinements aimed at minimizing unexpected fractures, particularly those attributable to cyclic fatigue, the susceptibility of NiTi files to fatigue failure remains a clinical concern [32]. In the current investigation, TN files recorded the highest cyclic fatigue resistance, followed by PTU and PTN. This may relate to the combination of TN’s slim-wire design, the parallelogram cross-sectional geometry, and proprietary post-machining gold heat treatment [33], which reorganizes the alloy microstructure, releases internal stresses, and promotes martensitic transformation at body temperature, resulting in exceptional flexibility and fatigue resistance [34]. On the other hand, PTU files incorporate a variable-angle parallelogram cross-section that modulates the cutting efficiency along the instrument length based on anticipated workload distribution and positions the mass eccentrically relative to the rotation axis, reducing stress accumulation during cutting [35–37]. Moreover, PTU finishers are fabricated from gold heat-treated NiTi exhibiting a combination of martensitic and austenitic phases, conferring both cyclic fatigue resistance and flexibility [38].

By contrast, PTN is manufactured from M-wire NiTi, a thermomechanically treated alloy with a proportionally higher austenitic content compared to TN and PTU, resulting in greater stiffness and increased susceptibility to cyclic fatigue [39]. Furthermore, PTN’s rectangular cross-section entails a greater metal mass, which may increase its susceptibility to fracture, as explained by Srikumar et al. [40]. Ramteke et al. [41] linked TN’s unique design to its superior fatigue performance. Similarly, Aranguren et al. [42] demonstrated TN’s statistically significant superiority over 16 of the 20 rotary systems evaluated, including PTU and PTN. Rocha et al. [26] reported significantly greater time to fracture for TN versus PTU, and Riyahi et al. [43] confirmed TN’s significant superiority over PTN in cyclic fatigue resistance.

It is pertinent to note that all files in the current study were size 25 but of differing tapers (8 for PTU, 4 for TN, 6 for PTN). Increased taper may negatively impact cyclic fatigue resistance, as reported by Faus et al. [44], which may partly account for TN’s improved cyclic resistance over PTU. In a comparison involving TN glide path instruments and PTU glide path files, Türk et al. [33] reported greater cyclic fatigue resistance for PTU, a finding that appears to conflict with the current results. However, this discrepancy is likely attributable to differences in instrument sizes used across studies.

The literature contains contradictory evidence regarding the effects of autoclave sterilization on NiTi files, reflecting heterogeneous study designs and outcomes [45]. In the present study, PTU and PTN were unaffected by multiple autoclaving cycles, whereas TN demonstrated a significant drop in cyclic fatigue resistance with increasing autoclave exposure. The lower metal mass may render TN more susceptible to microstructural changes caused by thermal sterilization [52]. Moreover, repeated autoclaving has been reported to alter the transformation temperatures in NiTi alloys, potentially shifting the martensite-to-austenite phase ratio and reducing flexibility and cyclic fatigue resistance [46]. However, the exact mechanism remains uncertain, as TN is manufactured using a proprietary thermomechanical treatment.

On the other hand, Stosic et al. [47] suggested that the M-wire technology, used in the production of PTN, involves a series of heat treatments that make it more elastic and resistant to fracture, even with multiple autoclaving cycles. Furthermore, PTU is subjected to a post-machining proprietary gold heat treatment that tends to stabilize the alloy’s microstructure during repeated autoclaving [48]. These findings align with those of Bardan et al. [51], who reported no autoclaving-related effect on cyclic fatigue resistance for PTU, and with Yahya et al. [49] and Mostafa et al. [50] who proposed that standard autoclave temperatures may be insufficient to induce microstructural alterations in NiTi alloys that would measurably affect cyclic fatigue resistance. Conversely, Stosic et al. [47] reported that autoclaving significantly elevated PTN’s cyclic fatigue resistance, contrary to the current findings. However, the potential adverse effects of repeated autoclaving on the mechanical performance of rotary instruments should be considered when determining the feasibility of their repeated reuse.

The surface topography of rotary files significantly influences their clinical performance [55]. Lopes et al. [15] established an inverse relationship between surface roughness and cyclic fatigue resistance, as crack nucleation in smooth files requires proportionally more time than in rough files, where surface defects provide readily available initiation sites for crack propagation [15]. In the present study, surface roughness measurements were obtained from a standardized area located 3 mm from the instrument tip, rather than from the entire instrument surface, to ensure the accuracy and reliability of the results. PTN was found to be significantly rougher than PTU, contradictory to the findings of Martins et al. [35]. TN demonstrated lower surface roughness than PTN, corroborating the observations of Merima et al. [35] and Parmar et al. [51]. The relative smoothness of TN may be related to its fabrication from a slender wire with a diameter of 0.8 mm [52].

Repeated autoclaving was associated with a significant increase in surface roughness of TN only. This contrasts with the findings of Ba-Hattab et al. [53], who reported a reduction in TN surface roughness following autoclaving. Furthermore, the current results are contradictory with those of Özdemir et al. [54], who documented a significant increase in PTN surface roughness after five autoclaving cycles, attributing this to micro-pitting of the alloy surface.

This study evaluated root canal dentine roughness after instrumentation with different file systems given its recognized relevance to endodontic treatment outcomes. Scanning electron microscopy (SEM) provides an accessible method for surface quality analysis [30]. SEM combined with ImageJ image analysis was selected in the present study to evaluate surface topographical changes because it provides high-resolution visualization of surface morphology and enables comparative quantitative image analysis [55]. Previous studies have demonstrated the applicability of ImageJ-based analysis for the assessment of surface characteristics [56]. Liu et al. [31] employed ImageJ analysis to evaluate surface roughness of dentine depending on SEM images.

In non-autoclaved files, PTN resulted in the highest dentine roughness in all dentine root thirds, while PTU produced the least dentine roughness in apical and middle regions and TN caused the lowest roughness in coronal regions. This is consistent with file roughness testing, in which PTN showed the highest surface roughness at zero autoclaving cycles. Also, PTN possesses an off-centered rectangular cross-section, which produces an asymmetric rotary motion [57]. Additionally, the PTN taper (0.06) may result in the removal of larger amounts of dentine [58]. 

Conversely, non-autoclaved TN produced smoother dentine surfaces in the coronal region, which might be attributed to its slim diameter and off-centered design aimed at preserving dentine [59]. Furthermore, TN has a taper of 0.04, which is lower than PTN and might result in less dentine removal. These results are consistent with the results of a study by Yanik and Ozel, who compared the shaping ability of TN and PTN using micro-computed tomography and found that the removed dentine by TN was much lower than that by PTN [60]. Also, PTU resulted in the least dentine roughness in the apical and middle thirds, which could be attributed to file geometry in which the center of mass is not aligned with the center of rotation, thus reducing the stress level during cutting. Also, the increased flexibility of the file due to heat treatment may allow the file instrumentation with a less aggressive engagement of dentine.

Repeated autocalving significantly changed dentine roughness for all three rotary file systems. In PTU, dentine roughness increased with increasing numbers of autoclaving cycles in most dentine regions. One possible explanation is that exposure to thermal changes during autoclaving might alter the file surface or cutting edges, which can affect cutting efficiency and produce more irregular dentine surfaces [61]. Moreover, previous studies showed that repeated autoclaving cycles can increase surface oxidation, surface wear and micro-pitting [25, 62]. These results are consistent with the results of the files’ surface roughness testing, which showed that in PTU, file roughness increased with an increase in the number of autoclaving cycles, although the increase at 5 cycles was not significant.

For TN, dentine roughness significantly increased after 10 cycles in the apical and coronal thirds. TN has a reduced metal mass so it is possible that exposure to heat during sterilization might have caused changes in the alloy differently than conventional heat-treated alloys [46]. On the other hand, dentine roughness decreased with PTN after exposure to different numbers of autoclaving cycles among several subgroups. Although the exact mechanism is unknown, this may be attributed to minor surface changes in the file caused by repeated sterilization, which could reduce cutting efficiency through subtle wear. Another explanation might be that the increase in the roughness of an endodontic file might increase the friction with dentine surface, which can decrease cutting efficiency and subsequently the amount of dentine removed by the file [63]. Similar results were reported by Elkady and Gad [17] where PTN-instrumented canals were similarly found to exhibit elevated dentine roughness.

In the current study, the middle third region exhibited the highest surface roughness among several subgroups. Root canal anatomy differs along its length. In the coronal root third, greater file contact and more effective cutting are expected due to the straighter canal morphology and wider diameter. On the other hand, in the apical third, restricted instrument engagement might occur due to the limited canal diameter and increased curvature. These anatomical variations could affect cutting angle, stresses produced during instrumentation, and file dentine contact [64, 65]. The middle third is a transitional region that might be exposed to different stress distributions affected by file taper and canal curvature, which might explain the increased roughness after instrumentation.

The study acknowledges several limitations inherent to its in vitro design. First, testing was performed on files after mechanical debridement subjected to autoclaving; however, several clinical factors could not be included, e.g., exposure to irrigants such as EDTA and sodium hypochlorite and intraoral temperature. Second, artificial canals cannot fully mimic the anatomical complexity, dentine interaction, and stress distribution encountered in natural root canals. Furthermore, the investigated file systems differed in taper and geometric design, which could result in the removal of different amounts of dentine and the generation of different lateral contact forces against the canal walls during instrumentation. Also, inter-specimen variability in dentine substrate might occur that could be attributable to differences in patient age, degree of sclerosis, and pre-existing restorative conditions.

SEM combined with ImageJ analysis has several limitations compared with established quantitative techniques such as profilometry and atomic force microscopy (AFM). Conventional SEM provides predominantly two-dimensional morphological information, whereas profilometry and AFM can provide quantitative measurements of surface topography, including height variations and three-dimensional roughness parameters [66]. However, to minimize variability, standardized magnification, image processing examiner blinding and selection of area of interest were used. Further validation of the SEM approach against AFM or profilometry using corresponding specimens is recommended to strengthen validity.

## Conclusion

Repeated autoclave sterilization adversely affected the cyclic fatigue resistance and surface roughness of TN files, while the cyclic fatigue resistance of PTU and PTN remained unaffected. However, their surface roughness increased for PTU and PTN after exposure to 10 and 5 cycles, respectively. Moreover, autoclaving caused an average increase in dentine roughness instrumented using PTU and TN. In contrast, for PTN, dentine roughness significantly decreased in most subgroups after autoclaving. The effect of repeated autoclaving in this study was dependent on the file-system type. Clinicians should consider file-specific responses to autoclaving when establishing sterilization and reuse protocols to optimize instrument safety and root canal surface quality.

## Data Availability

The datasets used and/or analyzed during the current study are available from the corresponding author on reasonable request.

## Abbreviations

AFM: Atomic force microscopy
ANOVA: Analysis of variance
CFR: Cyclic fatigue resistance
NCF: Number of cycles to fracture
NiTi: Nickel–titanium
PTN: ProTaper Next
PTU: ProTaper Ultimate
Ra: Average surface roughness
SD: Standard deviation
SEM: Scanning electron microscope / Scanning electron microscopy
SR: Surface roughness
TN: TruNatomy
TTF: Time to fracture
